# Somatic Mutations in Nuclear and Mitochondrial Genes of Mitochondrial Proteins in Primary and Recurrent Glioblastoma

**DOI:** 10.3390/ijms27041773

**Published:** 2026-02-12

**Authors:** Marton Tompa, Bence Galik, Peter Urban, Attila Gyenesei, Bernadette Kalman

**Affiliations:** 1 National Genomics Center, Szentagothai Research Center, University of Pecs, 20. Ifjusag Street, 7624 Pecs, Hungary; galik.bence@pte.hu (B.G.); urban.peter@pte.hu (P.U.); gyenesei.attila@pte.hu (A.G.); 2 Molecular Medicine, Markusovszky University Teaching Hospital, 5. Markusovszky Street, 9700 Szombathely, Hungary; 3 Office of the Dean, School of Medicine, University of Pecs, 12. Szigeti Street, 7624 Pecs, Hungary

**Keywords:** glioblastoma, mitochondrial DNA, nuclear-encoded mitochondrial protein genes, next generation sequencing

## Abstract

The accumulation of somatic mutations contributes to clonal evolution and biological properties of cancers. Acquired mutations in mitochondrial (mt)DNA have been studied, but with the exception of those in isocitrate dehydrogenase genes, no comprehensive assessment of mutations in nuclear mitochondrial genes has been reported in sequential glioblastoma (GBM). We obtained ten pairs of GBM samples at diagnosis (GBM-P) and at recurrence (GBM-R). Extracted DNA was subjected to whole exome and mtDNA sequencing. After filtering out germline variants, bioinformatics analysis was performed using a mitochondrial gene panel of 483 nuclear-encoded, and 37 mtDNA-encoded genes. Variant classification was performed using established clinical- and molecular criteria, integrating population-frequency data, bioinformatic predictions, functional evidence, segregation information, and curated entries from the Mitomap and ClinVar databases. Benign single nucleotide variants in mtDNA-encoded genes of *RNR1*, *RNR2*, *ATP6*, *CYB*, *CO2*, *TV*, *ATP8*, and *ND2* were detected, which changed little over time. However, three variants in *TI*, *ND5* and *ND1* with possible or likely pathogenic significance were found in the GBM-R samples. In contrast, pathogenic or likely pathogenic variants in 29 nuclear genes were found in GBM-P and GBM-R samples. Not only the overall number, but also the number of protein-truncating variants in nuclear genes increased over time. Conclusions: This study sheds light on the accumulation of mutations in nuclear genes of mitochondrial proteins in sequential GBM samples. As such variants may influence metabolic, proliferative and invasive properties as well as the necrotic propensity of the tumor, a comprehensive analysis of these genes merits further studies.

## 1. Introduction

Glioblastoma (GBM) represents the most malignant and the most aggressive form of gliomas. The fourth and fifth edition of the World Health Organization classification of central nervous system (CNS) tumors (WHO CNS4 and WHO CNS5) integrated molecular genetic- and genomic alterations into the histopathological characteristics [1,2]. This guideline has significantly transformed how we understand and diagnose brain tumors in the clinic. In the meantime, research efforts made great advancements in defining multi-OMICS features of these tumors both in bulk tissues and at single-cell levels, thereby paving the road for the development of precision therapies [3]. Our research team used next-generation sequencing-based approaches for defining epigenomic and genomic profiles of sequential GBM samples [4,5,6,7,8]. From a corollary study using the same GBM tissue pairs, here we report occurrences and evolution of somatic mitochondrial (mt)DNA and nuclear mitochondrial gene mutations, characteristics typically not embraced by large consortial studies on GBM.

Somatic mtDNA alterations accumulate in various cancers, including gliomas, and may contribute to cancer genesis and maintenance. About 60% of tumors may harbor mtDNA mutations affecting key mitochondrial functions including oxidative phosphorylation (OXPHOS) and ATP synthesis, free-radical production, protein synthesis or apoptosis regulation [9]. Impaired mitochondrial function affects GBM development, evolution and drug resistance [9]. Mutations have been frequently detected in the D-loop region in association with altered mtDNA replication, transcription, copy number variations (CNV), enhanced free-radical production and overall mitochondrial dysfunction [9,10,11]. However, missense mtDNA mutations with pathogenic significance have also been found within protein-coding regions [9]. More recently, Soon et al. [12] reported that 75% of gliomas harbor at least one somatic mtDNA mutation, 45% of which may be pathogenic. Most frequently, the respiratory chain complex I and IV genes carry nonsynonymous mtDNA mutations [12]. Their prognostic significance, however, remains less clear.

Sourty et al. [13] also assessed mtDNA copy numbers in GBM tumors from patients with different age of onset and found that this marker prognosticates disease outcome differently in the younger and older groups of patients [13]. Others, however, concluded that mitochondrial copy number changes, mutations and heteroplasmy presently have limited utility for screening and diagnosing GBM [11]. We found only a few publications reporting simultaneously somatic alterations in mtDNA and nuclear-encoded genes of mitochondrial proteins in GBM [14,15], but these were cross-sectional studies not including sequential samples.

Therefore, here we aim to present our mtDNA and nuclear-encoded mitochondrial gene exome sequencing data from formalin-fixed, paraffin-embedded (FFPE) primary and recurrent GBM (GBM-P and GBM-R) specimens of adult patients, in order to define if this approach can reveal new information relevant to GBM pathogenesis or shed more light on its biological properties. The samples used here were also subjects of our previous epigenomic and genomic studies [4,5,6,7,8].

## 2. Results

### 2.1. Comparisons of Somatic mtDNA Variants in GBM-P and GBM-R Samples

#### 2.1.1. mtDNA Variants in GBM-P Samples (Table 1)

D-loop region was not included in these analyses, only the gene-coding regions, because of the coverage by the probes in the applied kit. The four most common variants present in 10/10 of the GBM-P samples were in the ribosomal RNA (rRNA) coding genes of *RNR2* at position M:3106 (mean variant allele frequency or VAF: 96%) and *RNR1* at position M:750 (mean VAF: 98%) as well as in the protein-coding genes of *ATP6* at position M:8860 (mean VAF: 100%) and *CYB* at position M:15326 (mean VAF: 99%). As the *RNR1* M:750, *ATP6* M:8860 and *CYB* M:15326 variants are ancient polymorphisms present in all major haplogroups, it is highly likely that these are not somatic, but rather germline variants that escaped the germline filter. The in silico prediction of the *ATP6* variant is likely benign, while there is no functional prediction of the *RNR1*, *CYB* and *RNR2* variants in the Mitomap database (For detailed description of mtDNA variant classification see Materials and Methods section).

The second most common variant was in the overlapping *rRNA1* and *tRNA* phenylalanine coding *RNR1-TF* gene region at position M:311 (mean VAF: 52%) present in 9/10 samples, but also without in silico significance.

The third most common variant appeared in 5/10 samples in *RNR2* at M:2557 (mean VAF: 7%) without in silico prediction

The fourth most common variants were detected in *RNR2* at position M:2523 (mean VAF: 7%) and in *RNR1-TF* at position M:73 (mean VAF 100%) also without in silico prediction, but representing ancient polymorphisms.

The remaining variants appeared in single samples and included single nucleotide variants (SNVs in *TF* at M:621, *CO1* at M:7356 and M:7408, *ATP6-ATP8* at M:8557, *CYB* at M:15442, *CO2* at M:7830, *TV* at M:1619, *ATP8* at M:8381 and *ND2* at M:5298. The mean VAF values of these variants were either at or below 10% (*TV*, *TF* and *CO1*) or beyond 95% (*ATP6-ATP8*, *CYB*, *CO2*, *ATP8*, *ND2*). The in silico prediction of these variants is likely benign, with the exception of the *TF* variant, that has a possible benign prediction.

In addition to the variants listed above and in Table 1, we detected several mtDNA variants in single GBM-P samples, which have no in silico prediction and no entries in the Mitomap database (see the complete list in Supplementary Table S1A).

**Table 1:** Somatic mtDNA variants detected in GBM-P and GBM-R samples.

				GBM-P			GBM-R		
Gene (Position)	Sequence Ontology	Nucleotide Change	Amino Acid Change	Mean VAF	In Silico Prediction	Frequency in Samples	Mean VAF	In Silico Prediction	Frequency in Samples
*RNR2* (M:3106)	NE-CV	C/-	-	96%	-	10/10	96%	-	9/9
*RNR1* (M:750)	NE-CV	A/G	-	98%	-	10/10	99%	-	9/9
*ATP6* (M:8860)	MV	A/G	Thr112Ala	100%	LB	10/10	100%	LB	9/9
*CYB* (M:15326)	MV	A/G	Thr194Ala	99%	-	10/10	99%	-	8/9
Section between *RNR1-TF* genes (M:311)	US-GV	-/C	-	52%	-	9/10	51%	-	8/9
*RNR2* (M:2557)	NE-CV	C/T	-	7%	-	5/10	7%	-	5/9
*RNR2* (M:2523)	NE-CV	C/T	--	7%	-	4/10			
Section between *RNR1-TF* genes (M:73)	US-GV	A/G	-	100%	-	4/10	100%	-	4/9
TF (M:621)	NE-CV	A/C	-	5%	PB	1/10			
*CO1* (M:7356; M:7408)	MV	G/A; A/G	Val485Met; Tyr502Cys	6%; 6%	LB	1/10; 1/10			
Section between *ATP6-ATP8 genes* (M:8557)	MV, SV	G/A	Ala11Thr; Leu64=	97%	LB	1/10	97%	LB	1/9
*CYB* (M:15442)	SV	A/G	Leu232=	99%	LB	1/10			
*CO2* (M:7830)	MV	A/G	Arg82His	100%	LB	1/10	100%	LB	1/9
*TV* (M:1619)	NE-CV	C/T	-	10%	LB	1/10			
*ATP8* (M:8381)	MV	A/G	Thr6Ala	97%	LB	1/10	97%	LB	1/9
*ND2* (M:5298)	MV	A/G	Ile277Val	98%	LB	1/10	97%	LB	1/9
*ND2* (M:4794)	MV	G/A	Ala109Thr				14%	LB	1/9
*TI* (M:4268)	NE-CV	T/C	-				25%	PP	1/9
*ND5* (M:13028)	MV	C/T	Pro231Leu				9%	PP	1/9
*ND1* (M:3502)	MV	T/C	Ser66Pro				12%	LP	1/9

This table summarizes mtDNA SNVs identified in GBM-P and GBM-R samples. Variants are listed by genes and genomic positions (according to rCRS notation), their mean variant allele frequency (VAF), pathogenicity prediction by the in silico prediction tool of Mitomap, and per-sample frequency. Among the detected variants, four turned out to be ancient polymorphisms: *RNR1* (M:750), *ATP6* (M:8860), *CYB* (M:15326), and a section between *RNR1-TF* genes (M:73). NE-CV: Non-exon-coding variant; MV: Missense variant; US-GV: Upstream gene variant; SV: Synonymous variant. Likely benign (LB), Possible benign (PB), Possible pathogenic (PP), Likely pathogenic (LP).

In summary, none of the mtDNA SNVs detected in the GBM-P samples had pathogenic or likely pathogenic in silico prediction.

#### 2.1.2. mtDNA Variants in GBM-R Samples (Table 1)

Because one of the GBM-R tissue blocks left over from the previous clinical and molecular studies did not have sufficient quantity and quality of tumor tissue, this one GBM-R sample was excluded from the present mtDNA analyses. The most frequently detected variant present in 9/9 of samples included *RNR2* at M:3106 (mean VAF: 96%), *RNR1* at M:750 (mean VAF: 99%) and *ATP6* at M:8860 (mean VAF: 100%). As indicated above, the *RNR1* M:750 and the *ATP6* M:8860 variants are ancient variants appearing in all major haplogroups, and therefore, are likely present in the patients’ germline. In silico prediction is likely benign for the *ATP6* M:8860 variant, while no prediction is available for the other two variants.

The second most frequent variants were detected in 8/9 samples in *CYB* at M:15326 (mean VAF: 99%) and *RNR1-TF* at M:311 (mean VAF: 51%). Neither one of them has in silico prediction. The *CYB* variant also represents an ancient polymorphism present in all major haplogroups.

The third most common variant was found in 5/9 samples in *RNR2* at M:2557 (mean VAF: 7%), without in silico prediction.

The fourth most common variant appeared in 4/9 samples in *RNR1-TF* at M:73 (mean VAF: 100%), without in silico prediction.

The remaining variants appeared only in single samples, and included SNVs in *ATP6-ATP8* at M:8557, in *CO2* at M:7830, in *ATP8* at M:8381, in *ND2* at M:5298 and M:4794, in TI at M:4268, in *ND5* at M:13028 and in *ND1* at M:3502. The mean VAF values for these variants greatly varied with 97%, 100%, 97% and 98% in the former four genes. These variants are all SNVs with likely benign in silico prediction. The mean VAF values were 25%, 9% and 12% for the last three gene SNVs with possible pathogenic (*TI* at M:4268 and ND5 at M:13028) and likely pathogenic (*ND1* at M:3502) prediction. The likely benign *ND2* SNV at M:4794 had a mean VAF of 14%.

In addition to the variants listed above and in Table 1, we detected several mtDNA variants in single GBM-R samples, which have no in silico prediction and no entries in the Mitomap database (see the complete list in Supplementary Table S1A).

In summary, three of the mtDNA SNVs detected in the GBM-R samples may have functional significance based on in silico prediction of possible (*TI* at M:4268 and *ND5* at M:13028) or likely pathogenic (*ND1* at M:3502) classification.

Several of the variants with VAF values at or just below 100% are known haplogroup markers representing ancient germline polymorphisms that leaked through the germline filter. Variants with low VAF (<25%) may represent real somatic changes. Of note, most of the possible and likely benign or unclassified variants were detected in both GBM-P and GBM-R samples, further suggesting that they were not related to clonal evolution of the tumors.

#### 2.1.3. Comparison of mtDNA Variant Numbers in GBM-P and GBM-R Samples (Table 2)

Table 2 suggests no clear trend for the direction of changes, whether the numbers of variants are predominantly increasing or decreasing in the GBM-P and GBM-R samples, though the cohort is too small to draw a far-reaching conclusion. Equal numbers in one sample pair, decreasing numbers in three (the seventh pair was excluded from this comparison, since the GBM-R sample was missing) and increasing numbers in five sample pairs can be observed, when all detected mtDNA variants are taken into account. There seems to be no trend for any correlation between the direction of changes (in Table 2) and the T1–T2 recurrence time or overall survival (OS) (clinical data are provided in the Materials and Methods section). However, the most noteworthy variants are the ones with possible or likely pathogenic Mitomap classification and lower VAF values in the *TI*, *ND5* and *ND1* genes of the GBM-R samples (Table 1). These variants, detected in the GBM-R counterparts of sample pairs 8, 10 and 2, respectively, are likely somatic mutations related to clonal evolution of late tumors, which again, did not show any correlation with the T1–T2 recurrence time periods or OS. Overall, however, this analysis shows that the occurrence of biologically significant mtDNA variants during the evolution of GBM tumors are low. Nevertheless, the number of individually occurring single random variants with unlikely biological significance, e.g., synonymous mutations, is altogether higher and may be reviewed in the details in Supplementary Table S1A.

**Table 2:** Numbers of mtDNA variants detected in GBM-P and GBM-R samples.

Variant Number Change in GBM-P Versus GBM-R Samples		
GBM-P	GBM-R	Observed trend
First pair		
27	9	Decrease
Second pair		
9	10	Increase
Third pair		
23	8	Decrease
Fourth pair		
17	13	Decrease
Fifth pair		
9	9	Equal
Sixth pair		
18	20	Increase
Seventh pair		
6	0	Excluded
Eight pair		
14	15	Increase
Ninth pair		
8	12	Increase
Tenth pair		
5	8	Increase

This table shows the numbers of mtDNA variants detected in GBM-P and GBM-R samples.

### 2.2. Pathogenic and Likely Pathogenic Somatic Variants in Nuclear-Encoded Genes of Mitochondrial Proteins in GBM-P and GBM-R (Table 3)

In GBM-P samples, there are six missense SNVs, one nonsense SNV and five splice-site variants with pathogenic/likely pathogenic (P/LP) significance according to the ACMG classification and ClinVar database (For nuclear mitochondrial variant classification, see a detailed description in Materials and Methods section). In the GBM-R samples, there are eight missense SNVs, seven nonsense SNVs, two frameshift SNVs and six splice-site variants with P/LP significance. Also noteworthy is that there is only one protein-truncating variant in the GBM-P, while nine such variants are detected in the GBM-R group.

In six of the nine sample pairs, the numbers of somatic SNVs are increasing in the GBM-R compared to the GBM-P samples. Sample pair 2 had no somatic variants detected in either GBM-P or GBM-R. In two sample pairs (eighth and nineth pairs), the detected SNV numbers are decreasing. Sample pair 7 was excluded since the GBM-R sample was missing. While the sample size is too low to make far reaching conclusions, the trend of increasing P/LP variant numbers in nuclear-encoded mitochondrial genes is still suggestive during tumor evolution. Nevertheless, we could not see a reliable correlation between the direction of variant number changes in Table 3 and the T1-T2 recurrence time or OS in Table 4.

Overall, however, there seems to be a trend for the rise in damaging variants over time, likely affecting mitochondrial bioenergetics (*SDHB*, *SDHAF2*, *FH*, etc.), replication (*POLG*, *TWNK*, etc.), enzyme function (*CPT1A*, *CPT2*, *CYP24A1*, *FA2H*, etc.) or intracellular transport (*SLC25A4*, *SLC25A13*, *SLC22A5*, etc.). Allelic frequency of the variants vary between 15% and 75% in GBM-P samples, and between 16% and 39% in GBM-R samples, suggesting that the proportion of functionally significant somatic mutations may reach a biological threshold affecting cell metabolism, survival and propagation.

Finally, it is also notable that the acquired genetic variants with P/LP significance detected in the primary tumors persisted in none of the recurrent tumors, where, however, novel somatic variants with P/LP significance occurred. This observation suggests that somatic nuclear-encoded mitochondrial gene variants with P/LP significance frequently appear and disappear during clonal tumor evolution, likely related to the metabolic properties, invasiveness and necrotic preponderance of GBM. For details of all detected somatic nuclear-encoded mitochondrial gene variants see Supplementary Table S1B.

**Table 3:** Pathogenic and likely pathogenic somatic nuclear-encoded mitochondrial gene mutations in GBM-P and GBM-R samples.

GBM-P				GBM-R			
VAF	Gene	P/LP	Variant	VAF	Gene	P/LP	Variant
First pair							
				15%	*SLC25A13*	P	c.111del; p.Asn38fs
				15%	*FA2H*	P	c.117C>A; p.Phe39Leu
Second pair							
NA							
Third pair							
				32%	*WFS1*	P	c.2254G>T; p.Glu752Ter
				26%	*SDHAF2*	LP	c.260+1G>C; p.?
Fourth pair							
				20%	*SEPSECS*	P	c.1655_1656del; p.Lys552fs
				17%	*SLC22A5*	LP	c.914C>T; p.Pro305Leu
				29%	*SLC22A5*	P	c.1658+1G>A; p.?
				22%	*PLA2G6*	LP	c.120-1G>T; p.?
Fifth pair							
17%	*SLC22A5*	LP	c.914C>T; p.Pro305Leu	39%	*SDHB*	P	c.441T>G; p.Tyr147Ter
				28%	*DIAPH1*	P	c.3139C>T; p.Gln1047Ter
				23%	*POLG*	LP	c.2841A>C; p.Lys947Asn
				16%	*COQ8B*	P	c.835C>T; p.Arg279Trp
Sixth pair							
				22%	*CPT2*	P	c.215T>A; p.Leu72Ter
				19%	*AARS2*	P	c.595C>T; p.Arg199Cys
				25%	*PDHA1*	P	c.904C>T; p.Arg302Cys
Seventh pair							
34%	*BCS1L*	P	c.826C>T; p.Gln276Ter	31%	*SLC25A4*	P	c.111+1G>A; p.?
75%	*SLC52A2*	P	c.1258G>A; p.Ala420Thr				
48%	*STXBP1*	P	c.1419+5G>A; p.?				
26%	*TWNK*	LP	c.1232C>T; p.Thr411Met				
Eight pair							
15%	*FH*	P	c.1391-1G>T; p.?	32%	*PC*	LP	c.1826-1G>C; p.?
15%	*FH*	P	c.1391-2A>G; p.?	24%	*POLG*	P	c.178C>T; p.Gln60Ter
17%	*ACADS*	LP	c.1086+1G>A; p.?	19%	*ACADVL*	LP	c.740A>C; p.Lys247Thr
26%	*LONP1*	LP	c.880C>T; p.Arg294Trp				
Ninth pair							
31%	*MFN2*	P	c.1127T>G; p.Met376Arg	33%	*CPT1A*	P	c.1367C>A; p.Ser456Ter
59%	*SDHB*	LP	c.540+1G>A; p.?	35%	*TANGO2*	P	c.262C>T; p.Arg88Ter
34%	*CYP24A1*	P	c.1186C>T; p.Arg396Trp				
Tenth pair							
				24%	*GDAP1*	LP	c.-67A>G; p.Lys39Arg
				16%	*GDAP1*	LP	c.117+1G>A; p.?

This table displays the variant allele frequency (VAF), gene name, pathogenic (P) or likely pathogenic (LP) classification, and the variant itself in GBM-P and GBM-R samples.

**Table 4:** Clinical characteristics of patients.

GBM Designation	Gender	Age at Onset (Years)	Treatment	T1–T2 (Weeks)	Overall Survival (Weeks)
GBM 1	man	61	Surgery + irradiation + TMZ	49	60
GBM 2	man	39	Surgery + irradiation + TMZ	40	-
GBM 3	man	62	Surgery + irradiation + TMZ	58	62
GBM 4	woman	61	Surgery + irradiation + TMZ	31	54
GBM 5	man	66	Surgery + irradiation + TMZ	56	-
GBM 6	woman	53	Surgery + irradiation + TMZ	55	69
GBM 7	woman	63	Surgery + irradiation	30	43
GBM 8	woman	45	Surgery + irradiation + TMZ	143	170
GBM 9	man	43	Surgery + irradiation + TMZ	135	192
GBM 10	woman	56	AVAGLIO clinical study (STUPP + bevacizumab/placebo)	199	288

This table shows GBM designation, patients gender, age at onset, treatment modality, T1–T2 and overall survival time (OS). T1–T2 recurrence time is calculated from the dates of first and second surgery (weeks), and OS is calculated from the dates of first surgery and death (when the time of death was available). This table has been modified from Table 4 published in the International Journal of Molecular Sciences, DOI: https://doi.org/10.3390/ijms25147564 [8].

## 3. Discussion

Mitochondria are central regulators of bioenergetics, metabolism, and survival. These cytoplasmic organelles provide energy for cells by oxidative phosphorylation (OXPHOS) and adenosine triphosphate (ATP) synthesis, but are also involved in the regulation of programmed cell death and necrosis, stress responses, Ca homeostasis, mitochondrial quality-control and expression of nuclear genes by a retrograde signaling [16,17,18]. The mitochondrial architecture is highly dynamic and exhibits marked cell type-specific differences in both structure and proteome, allowing the organelle to meet distinct metabolic demands [19]. Reflecting these central cellular roles, mitochondrial dysfunction is implicated in a wide spectrum of diseases, including metabolic disorders, cardiomyopathies, neurodegeneration and cancer [20].

**Figure 1:**
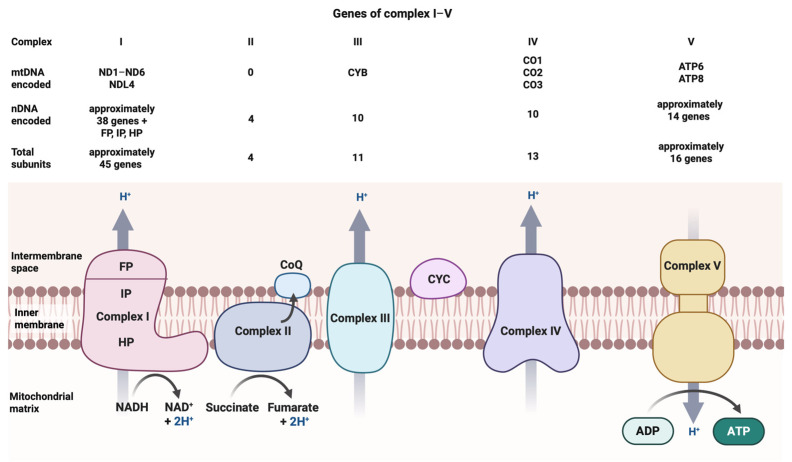
Components of the mitochondrial electron transport chain serving OXPHOS. The figure schematically depicts the five enzyme complexes composing the mitochondrial electron transport chain in the lower part, and shows numbers of mtDNA and genomic DNA-encoded subunits of these proteins in the upper part. Note that with the exception of Complex II (that is exclusively encoded by 4 nuclear genes), the remaining enzyme complexes have dual genetic origins. This schematic figure is adapted from Figure 1 in the publication by Kalman et al. [21].

Mitochondrial proteins are encoded by genes in the mtDNA and the nuclear DNA. In addition to the regulatory D-loop region, mtDNA has 37 genes, including 13 protein-encoding, 22 transfer RNA and 2 ribosomal RNA genes. The genes of five OXPHOS enzyme complexes have dual mtDNA and nuclear DNA origins with the exception of Complex II that is exclusively encoded by nuclear genes (Figure 1). The 13 mtDNA-encoded proteins are all included in OXPHOS enzyme complexes I, III, IV and V.

**Figure 2:**
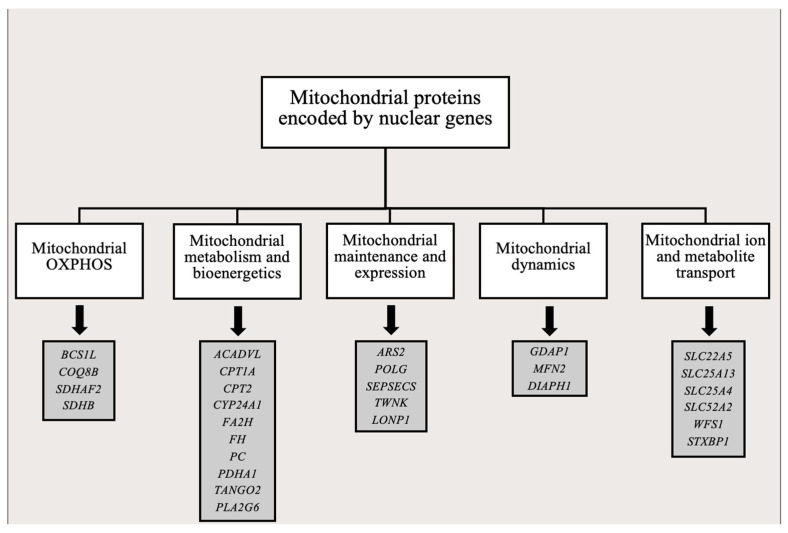
Functional groups of mitochondrial proteins encoded by nuclear genes. Boxes in the figure show functional classification of nuclear-encoded mitochondrial genes with acquired P/LP variants in GBM-P and GBM-R samples.

The estimated number of nuclear-encoded mitochondrial proteins is close to 1500. Besides composing OXPHOS subunits or cofactors involved in ATP synthesis, these gene products play important roles in amino acid and nucleotide production, mtDNA replication and transcription, mitochondrial protein biogenesis and turnover, cellular signaling, membrane dynamics, stress responses and apoptosis regulation (Figure 2) [22]. Morgenstein et al. [23] reported that approximately 31% of mitochondrial proteins contribute to metabolic pathways, and 22% are associated with regulation of membrane dynamics, protein biogenesis, signaling and stress responses [22,23]. The function of about 14% of mitochondrial proteins remains less defined.

Pathogenic variants affecting components of the electron transport chain (ETC) reduce ATP production and increase reactive oxygen species (ROS), while tricarboxylic acid (TCA) cycle defects limit nicotinamide adenine dinucleotide (NADH) and flavin adenine dinucleotide (FADH_2_) supply, reinforcing energetic and oxidative stress. Structural alterations, including fusion–fission imbalance and cristae remodeling, destabilize respiratory supercomplexes and impair OXPHOS. Disrupted BCL2-associated agonist of cell death (BAD)–glucokinase and BCL2-associated X protein (BAX) signaling uncouples glycolysis from respiration, while the loss of the mitochondrial membrane potential disrupts calcium (Ca^2+^) handling and endoplasmic reticulum (ER)–mitochondria crosstalk. Collectively, these defects weaken B-cell lymphoma 2 (Bcl-2)/B-cell lymphoma-extra large (Bcl-xL) survival pathways, enhance chaperone-mediated autophagy (CMA) dependent degradation of enzymes (e.g., HK2), and increase cellular stress sensitivity [24,25,26].

Due to the central role of mitochondria in cellular bioenergetics, redox balance, and apoptotic signaling, mitochondrial dysfunction is closely linked to the development of cancer biology and treatment resistance, and specifically to those of GBM [9,27]. A well-described metabolic characteristic of cancers is oxidative glycolysis or Warburg effect, when energy is predominantly produced by glycolysis even in the presence of oxygen. This process leads to lactic acid fermentation in the cytosol, rather than oxidative phosphorylation in mitochondria. While this is an inefficient way of producing ATP, oxidative glycolysis allows tumors cells to survive in a hypoxic environment, producing important metabolites for rapid proliferation and promotes tumor invasion. In addition to an altered glucose metabolism, changes in amino acid and lipid metabolism, impaired cell death/survival regulation, increased mitochondrial biogenesis, upregulation of mitochondrial fission, and altered mitophagy have all been found and linked to the accumulation of acquired mitochondrial protein variants in cancers [17].

According to data from the International Cancer Genome Consortium (ICGC) and The Cancer Genome Atlas (TCGA), mtDNA mutations are present in approximately 60% of solid tumors. These acquired somatic mutations are characterized by heteroplasmy (the coexistence of mutant and wild-type mtDNA species within a single cell) and believed to occur early in carcinogenesis as essential events in neoplastic transformation that also involves alterations in OXPHOS or mitochondrial ribosomal function, impaired ATP production, and increased oxidative stress [28,29]. Pathogenic mtDNA mutations and deletions typically result in a clinical phenotype only when they exceed a threshold level, approximately 80% for point mutations and 60% for deletions, within affected tissues [30,31]. While such heteroplasmy levels are well-described in classical mitochondrial disorders, their relevance in cancer, particularly GBM, remains unclear. Indeed, the biological significance of somatic mtDNA variants in GBM has not yet been systematically explored in experimental systems. A few recent studies have examined the impact of mtDNA copy number variation in GBM, but their findings are also somewhat contradictory. While a low mtDNA copy number was linked to therapy resistance and stemness features, a higher copy number was associated with improved prognosis and overall survival [32,33]. Of note, numerous studies analyzed the accumulation of somatic genomic variants in GBM using array-based or sequencing methodologies; nevertheless, we found only a few reports regarding the roles of such variants in nuclear-encoded mitochondrial genes in GBM [14,15] and none in sequential GBM.

Therefore, here we simultaneously analyzed mtDNA and nuclear DNA-encoded variants of mitochondrial genes in paired GBM-P and GBM-R samples to assess their frequency, distribution, and potential functional relevance relying on databases such as Mitomat and ClinVar, along with ACMG autoclassification included in our analysis software, VarSeq (for details, see Section 4). The ACMG autoclassification is based on population-frequency data, bioinformatic predictions, functional studies, segregation information, the published literature and clinical observations when sorting variants into benign, likely benign, variant of uncertain significance (VUS), likely pathogenic and pathogenic categories.

We detected relatively few somatic mtDNA variants with no consistent directional changes in total mtDNA variant counts when GBM-P and GBM-R samples were compared (Table 1 and Table 2, Supplementary Table S1A). According to the Mitomap database, most filtered variants, particularly those with high VAF values, were ancient haplogroup polymorphisms, likely representing germline variants that passed through the germline filtering. This observation aligns with previous GBM mtDNA studies highlighting the difficulty of distinguishing true somatic mutations from inherited polymorphisms. Kirches et al. [34] performed direct sequence comparisons between a GBM tumors and corresponding blood samples, and found that many mtDNA variants initially presumed to be somatic were in fact ancient haplogroup-defining germline polymorphisms [34]. These observations also align with the findings of Vega et al. [35], who reported that cells harboring intact or minimally mutated mtDNA are preferentially selected and retained in a highly proliferative environment. Only a small fraction of mtDNA variants may have functional significance and contribute directly to tumorigenesis, with the majority representing known polymorphisms [35]. Presumably variants with a low level of VAF (<30%) can be considered true somatic variants.

In our cohort, only three mtDNA variants (*TI* M:4268, *ND5* M:13028, *ND1* M:3502) had this lower level of VAF. These three variants were only identified in GBM-R samples, and were associated with possible or likely pathogenic significance according to the Mitomap in silico prediction tool (Table 1). This observation is supported by previous studies showing that Complex I genes, particularly *ND5*, are frequent mutational hotspots in GBM [14]. The *TI* M:4268 variant has been associated with defective mitochondrial processing of tRNA isoleucine [36] which may impair protein synthesis and compromise mitochondrial function in metabolically demanding tumor cells. *ND1* and *ND5* encode core subunits of Complex I. Mutations in these genes are known to impair OXPHOS, elevate ROS levels, and promote metabolic rewiring in cells, thereby supporting tumor growth and leading to therapy resistance [14,37]. tRNA gene mutations can reduce the translational efficiency of mitochondrial-encoded proteins, further destabilizing cellular energy balance and redox homeostasis. The detection of these low-heteroplasmy variants in recurrent tumors suggests clonal selection or adaptive shifts in mitochondrial function under therapeutic pressure. Such changes may promote an overall metabolic state that supports GBM survival, treatment resistance and progression. Although few somatic mtDNA variants were found, their presence in key Complex I subunits and tRNA regions may have significant implications for tumor evolution. Overall, most mtDNA mutations were nucleotide transitions resulting in missense mutations and showed an unequal distribution between the heavy strand and light strand, with a predominance in the heavy strand, a pattern previously associated with modifier effects in GBM tumorigenesis [38]. With the exclusion of ancient polymorphisms, we identified eight missense, one synonymous, and seven non-coding or upstream variants in the GBM-P cohort, compared to seven missense and four non-coding or upstream variants in the GBM-R cohort. This comparison showed a persistence of missense variants, while several non-coding changes were lost, suggesting that protein-altering variants may be preferentially retained during recurrence. Altogether, these findings suggest that mtDNA mutations in GBM are predominantly missense, with most classified as likely or possibly benign by in silico prediction tools, indicating they are unlikely to be major drivers of tumor evolution or recurrence. Nevertheless, it is also important to note that most somatic mtDNA point mutations in GBM were reported within the D-loop region [38], a known mutational hotspot during population evolution, aging, and oncogenesis. For technical reasons, we omitted this region from our study (as our kit had probes only for gene-coding mtDNA regions), and focused on tRNA, rRNA, and protein-coding genes of mtDNA, which are presumed to have more direct biological effects [9,38].

While mtDNA alterations appeared relatively limited in number and impact, the analysis of nuclear-encoded mitochondrial genes revealed a different picture in our sample cohort. Here, we focused on and presented only ClinVar-based P/LP variants in these genes and omitted from the presentation benign and VUS variants (details of all nuclear mitochondrial gene variants can be seen in the Supplementary Table S1B). To our surprise, an array of P/LP variants were detected in nuclear mitochondrial genes, a finding that has not been reported in previous whole genome or exome next-generation sequencing (NGS) studies in GBM (Table 3). In our prior WES study involving the same sample pairs, we used different bioinformatics filtering and performed a glioma panel analysis which lacked the mitochondrial target genes, explaining why the nuclear-encoded mitochondrial genes were not then reported [8]. From a bioinformatics perspective, the key distinction between the previous glioma panel and the present mitochondrial panel study lies in the use of population and COSMIC databases for variant filtering in the former analysis. From the perspective of panel components, only a limited set of genes—*DCC*, *FH*, *PDK1*, *SDHA*, *SDHAF2*, *SDHB*, *SDHC*, and *SDHD*—were included in both the glioma and the mitochondrial gene panels. Among these, only *SDHAF2* and *SDHB* harbored likely pathogenic or pathogenic variants according to the ACMG classification and ClinVar entries in the glioma panel analysis.

Why other NGS-based analyses missed detecting variants in nuclear genes of mitochondrial proteins may be related to a more stringent filtering or the lack of sufficient coverage and sensitivity to detect variants with lower VAF in these genes. The VAF values for most of our detected P/LP variants were below 30%. Alternatively, the use of a dedicated gene panel in our analyses might have enriched and enhanced prominence of mitochondrial variants in our samples. Our data interestingly suggest that nuclear-encoded mitochondrial gene variants are present in both early and late GBM, but generally do not persist over time, consistent with a high clonal turnover and a dynamic metabolic adaptation of tumor cells.

The P/LP classification used here follows the ACMG guidelines and links the identified variants to inherited diseases associated with proven impaired biological function of the encoded mitochondrial proteins. Cancer is not a primary (inherited) mitochondrial disorder, but the acquired genetic alterations during oncogenesis and gliomagenesis may impair proteins and biological pathways also altered in mitochondrial diseases [17]. Therefore, an established functional significance of the detected GBM variants and a biological dysfunction of affected proteins may be assumed. Many of these affected proteins play central roles in energy metabolism, mitochondrial genome maintenance and expression, protein folding and quality control, signaling, and membrane dynamics [19]. The observed variants may therefore contribute to the functional rewiring of mitochondrial processes during tumor evolution (Figure 2). Based on the comparisons of GBM-P and GBM-R profiles, these mutations may appear and disappear during disease progression, likely reflecting the selective pressure imposed by high metabolic demand, rapid proliferation, and necrosis. Notably, P/LP variants in genes such as *SDHB*, *SDHAF2*, *POLG*, *TWNK*, the *SLC* family genes, and *FH*, along with the enrichment of truncating variants at recurrence, support the notion that nuclear-encoded mitochondrial protein dysfunction is a dynamic driver of GBM evolution [3,9,39]. Functionally, turnover and recurrent acquisition of these variants may reflect shifting metabolic demands under hypoxia, proliferative pressure and therapy, selecting for tumor clones capable of fine-tuning OXPHOS vs. oxidative glycolysis balance, ROS handling and mitochondrial quality control [9]. The increase in truncating variants at GBM-R suggests stronger selection for broader pathway changes rather than small effects from single missense mutations.

In summary, this study provides insight into characteristic genomic changes and dynamics in paired primary and recurrent GBM samples. A strength of our approach is the use of deep bioinformatic analyses, which enhanced the detection of relevant variants and demonstrated the potential diagnostic value of NGS in individual cases. Limitations include the use of FFPE-derived DNA, which increased the risk of artifact detection despite rigorous filtering. The alkylating agent temozolomide is also mutagenic, which might have affected the mutational profiles of the GBM-R samples. Further, the small cohort size constrains the identification of novel variants, particularly of the recurrent variants, as well as the interpretation of findings. Due to the short survival of patients with GBM, the numbers of recurrent sample pairs were low to start with, and after quantitative and qualitative evaluation of the samples left over from prior clinical and research work up, the sample numbers further dropped. The targeted gene panel focused on known genes and pathways, but the immediate therapeutic application of the findings remains limited. As the occurrence of somatic mutations appears largely stochastic, it is somewhat difficult to perform clinically relevant in vitro functional studies. Therefore, in this small study we relied on ClinVar- and Mitomap-based classification to support the interpretation of their potential pathogenicity. How these variants with pathogenic and likely pathogenic significance specifically affect GBM tumor growth, merits further studies.

Our study revealed several new findings including a contrast between mtDNA and nuclear-encoded mitochondrial variants in GBM. Somatic mtDNA mutations were sparse and functionally questionable (with the exception of a few late-appearing P and LP variants), thereby suggesting limited tolerance for OXPHOS disruption in mtDNA-encoded proteins. In contrast, nuclear-encoded mitochondrial genes showed a broader and more dynamic spectrum of pathogenic variants, particularly truncating events, reflecting metabolic selection and clonal turnover during progression and therapy. While some mtDNA mutations affecting OXPHOS may contribute to therapy resistance, their overall functional role remains unclear, limiting their prognostic utility. By comparison, the greater adaptability of nuclear mitochondrial alterations underscores their potential as key modulators of tumor metabolism. Detection of recurrent somatic variants in large GBM cohorts, and integration of nuclear and mitochondrial genomic data with metabolic profiling will be essential to identify actionable vulnerabilities and guide network-level therapeutic strategies.

## 4. Materials and Methods

### 4.1. Subjects of the Study and Samples

Surgically excised FFPE GBM specimens were obtained in the period of 2003 and 2016 at the Department of Pathology, University of Pecs (UP). Blocks left over from clinicopathological evaluation were subjects of the present analyses. The study was approved (Number: 7517 PTE 2018) by the Regional Clinical Research Ethics Committee of the UP. After histological quality screening and DNA quantitative and qualitative assessments, ten pairs of immunehistologically proven isocitrate dehydrogenase (IDH)-1 R132H mutation negative initial (GBM-P) and recurrent (GBM-R) tumors from patients with late onset disease [age range: 39–66 years] were included. With the exception of one patient (GBM 7), who only had surgery and irradiation (prior to the approval of the Stupp protocol), all patients were treated with surgery, radiation therapy and temozolomide medication [40]. The clinical characteristics of patients are shown in Table 4. The diagnosis of primary (de novo) GBM was established based on standard clinical and histopathological criteria at the time when the study was initiated [1] and subsequently upgraded according to the 2021 WHO CNS5 criteria [2]. Thus, the selected IDH-1 R132H wild-type specimens had the histological features of high mitotic index, microvascular proliferation, necrosis and atypia, while also having the molecular characteristics of GBM according to the WHO CNS5 criteria. Whole exome sequencing confirmed that these samples were all *IDH1/2* wild-type. Of the 10 GBM-P, 6 had EGFR amplification, 2 homozygous and 1 heterozygous *CDKN2A* deletions. Of the 10 GBM-R samples, 7 had *EGFR* amplification, 2 homozygous and 3 heterozygous 10 *CDKN2A* deletions. Targeted sequencing revealed that eight of the GBM-P and nine of the GBM-R samples carried *TERT* promoter mutation (for details see Reference [8]). Primary tumor specimens were surgically obtained before, and recurrent specimens after, chemoradiation. As these samples were used in previous studies, details of sample characteristics have been reported [8].

### 4.2. Sample Preparation and Quality Check

DNA was isolated from FFPE tissue sections received from the Department of Pathology of UP. The samples were deparaffinized with xylene, and after washing with alcohol, the QIAamp DNA FFPE Tissue Kit (Qiagen^®^, Hilden, Germany) was used for the isolation of DNA. In the final step, DNA was eluted from the columns in 50 µL buffer and stored at −20 °C until further use. The concentration of the eluates was determined using the Qubit™ 1X dsDNA HS Assay Kit on a Qubit 4 Fluorimeter (Invitrogen, Carlsbad, CA, USA). Fragment analysis of the eluted DNA was performed by using the Agilent Genomic DNA Genomic DNA ScreenTape Assay kit on an Agilent 4200 TapeStation System (Agilent Technologies, Santa Clara, CA, USA). In the primary samples, the average rate was 50.34 ± 13.91% SD between 200 and 2000 bp fragments, while the average rate was 36.76 ± 8.75% between 2000 and 60,000 bp fragments. In the recurrent samples, the average rate was 58.13 ± 11.75% SD between 200 and 2000 bp fragments, while the average rate was 29.61 ± 9.12% between 2000 and 60,000 bp fragments [8]. Because of the fragmentation of DNA isolated from FFPE blocks, we attempted no analyses of copy number variations in this study.

### 4.3. Library Preparation

The mitochondrial (mt)DNA library preparation was conducted using DNA Prep with Enrichment (Illumina Inc., San Diego, CA, USA). Briefly, 100 ng FFPE DNA was tagmented, amplified and purified. Target capture was performed using the Twist Mitochondrial probes (Twist BioScience, South San Francisco, CA, USA). The nuclear (n)DNA library preparation was performed using the Sure Select Human All Exon V7 kit (Agilent Technologies, Santa Clara, CA, USA). Briefly, 50 ng DNA was enzymatically fragmented, end prepped, adaptor ligated and amplified. Hybridization was performed using Human All Exon V7 probes (Agilent Technologies, Santa Clara, CA, USA). The captured libraries were assessed using TapeStation 4200 and Qubit 3.0 (Invitrogen, Carlsbad, CA, USA). The mtDNA libraries were sequenced on an Illumina NovaSeq 6000 machine as part of our WES analyses (Illumina Inc., San Diego, CA, USA) for 2 × 150 paired end reads (see details [8]).

### 4.4. Bioinformatics for the Evaluation of mtDNA and nDNA Sequences

MtDNA and nDNA raw FASTQ files were generated, including basecalling and demultiplexing, by using bcl2fastq (v2.20.0.422) [41] on a local HPC cluster. Raw data quality was assessed with FastQC (v0.11.9) [42]. Subsequently, datasets were filtered and adapters, as well as low-quality bases and reads, were removed using fastp (v0.21.0) [42]. Clean, high-quality sequences were aligned to the human reference genome (GRCh37) in the case of nDNA, and to the Cambridge Reference Sequence (rCRS) of the Human Mitochondrial DNA (GenBank sequence: NC_012920) in case of mtDNA, using the bwa mem algorithm (v0.7.17) [43]. Default parameters were applied for chromosomal alignments, while specific parameters were used for mtDNA sequences (params: -M -K 100,000,000 -p -v 3 -Y).

SAM/BAM file modifications (e.g., sorting, adding read group information, indexing) were performed using various Picard Tools (v2.23.3) subcommands [44]. Mapping statistics and coverage metrics for both the whole exome and mtDNA were calculated using Picard Tools (v2.23.3) [44] and Qualimap (v2.2.1) bamqc [45], respectively. Before variant calling, base quality scores were recalibrated with the GATK Base Quality Score Recalibration (BQSR) module (v4.1.4.1) [46]. Somatic mtDNA single-nucleotide variants (SNVs) and short insertions and deletions (INDELs) were identified using the LoFreq algorithm [46], which was run in mitochondrial mode for mtDNA variant detection [47]. Somatic nDNA SNVs and INDELs were identified by GATK Mutect2 algorithm [46]. Finally, raw variants were pre-filtered by the GATK FilterMutectCalls module [46]. The components of the mitochondrial gene panel including the mitochondrial- and nuclear-encoded mitochondrial protein genes used in the analyses are shown in Supplementary Table S1C.

For mitochondrial and nuclear variant evaluation, the VarSeq software (v2.6.0; Golden Helix, Inc., Bozeman, MT, USA) was used for annotation and filtering of SNVs and INDELs. MtDNA and nDNA sequences from GBM pairs were uploaded as individual singletons. Only PASS-tagged variants were retained using the Low-Frequency Mitochondrial (mtDNA) Variant Caller and GATK FilterMutectCalls. Filtering criteria included a read depth ≥ 50 in both samples, while variant allele frequency (VAF) thresholds were ≥5% for mitochondrial variants and ≥15% for nuclear variants. Variants with entries in dbSNP (dbSNP Common 155, NCBI) were excluded. MtDNA variants with missing or ≤4 HRUN tag values (homopolymer length to the right of the reported indel position) were retained and a targeted gene panel (in Supplementary Table S1C) was used to focus on nuclear genes encoding mitochondrial proteins. As a final filtering step, mtDNA variants affecting both coding genes and short intergenic regions between genes were included for evaluation.

MtDNA variants identified by VarSeq were classified based on entries and in silico predictions in the MITOMAP database (https://www.mitomap.org/MITOMAP, accessed on 14 January 2026). nDNA variants identified by VarSeq were assessed according to the ACMG guidelines and the VarSeq’s autoclassification framework, which integrates population-frequency data, bioinformatic predictions, functional studies, segregation information, the published literature, clinical observations, and ClinVar entries (https://www.ncbi.nlm.nih.gov/clinvar/, accessed on 14 January 2026). Both the ACMG guideline and VarSeq autoclassification sort variants into benign, likely benign, VUS, likely pathogenic, and pathogenic categories. Most variants classified as pathogenic or likely pathogenic in the databases are supported by functional data in the literature. Variants assessed by VarSeq autoclassification, were cross-referenced by data in the ClinVar database.

## Data Availability

Raw data are available at the European Nucleotide Archive (ENA) under PRJEB63610 accession.

## Supplementary Materials

The following supporting information can be downloaded at https://www.mdpi.com/article/10.3390/ijms27041773/s1. Reference [48] is cited in the Supplementary Materials.

## Abbreviations

ACMG	American College of Medical Genetics and Genomics
BAD	BCL2-associated agonist of cell death
BAX	BCL2-associated X protein
Bcl-2	B-cell lymphoma 2
Bcl-xL	B-cell lymphoma-extra large
CMA	chaperone-mediated autophagy
CNV	central nervous system
CNV	copy number variations
ER	endoplasmic reticulum
ETC	electron transport chain
FADH	flavin adenine dinucleotide
FFPE	formalin-fixed, paraffin-embedded
GBM	glioblastoma
GBM-P	primary glioblastoma
GBM-R	recurrent glioblastoma
ICGC	International Cancer Genome Consortium
IDH	isocitrate dehydrogenase
LP	likely pathogenic
mtDNA	mitochondrial DNA
NADH	nicotinamide adenine dinucleotide
NGS	next-generation sequencing
OXPHOS	oxidative phosphorylation
P	pathogenic
rCRS	revised Cambridge Reference Sequence
ROS	reactive oxygen species
rRNA	ribosomal RNA
SNV	included single nucleotide variant
TCA	tricarboxylic acid
TCGA	The Cancer Genome Atlas
UP	University of Pecs
VAF	variant allele frequency
WHO	World Health Organization
